# Flea Communities on Small Rodents in Eastern Poland

**DOI:** 10.3390/insects11120894

**Published:** 2020-12-18

**Authors:** Zbigniew Zając, Joanna Kulisz, Aneta Woźniak

**Affiliations:** Chair and Department of Biology and Parasitology, Medical University of Lublin, Radziwiłłowska 11 St., 20-080 Lublin, Poland; joanna.kulisz@umlub.pl (J.K.); aneta.wozniak@umlub.pl (A.W.)

**Keywords:** fleas, rodents, *Ctenophthalmus agyrtes*, *Ctenophthalmus assimilis*, *Hystrichopsylla talpae*, *Nosopsyllus fasciatus*

## Abstract

**Simple Summary:**

Fleas are obligatory, secondarily wingless, hematophagous insects living all over the world. They colonize a variety of habitats from wet tropical forests to semi-arid and desert areas. Adult individuals feed mainly on small mammals, and less often on birds. The aim of the present study was to explore the fauna of fleas and their broad-sense behavior in eastern Poland. Rodents, which are widely recognized as one of the preferred hosts of these insects, were caught to carry out the study. The results show that, regardless of the ecological habitat type, the striped field mouse *Apodemus agrarius* was the most frequently captured rodent species, and the *Ctenophthalmus agyrtes* flea species was collected most frequently. Moreover, rhythms in the seasonal activity of fleas, with a peak in summer months, were noted.

**Abstract:**

Fleas are hematophagous insects infesting mainly small mammals and, less frequently, birds. With their wide range of potential hosts, fleas play a significant role in the circulation of pathogens in nature. Depending on the species, they can be vectors for viruses, bacteria, rickettsiae, and protozoa and a host for some larval forms of tapeworm species. The aim of this study was to determine the species composition of fleas and their small rodent host preferences in eastern Poland. Animals were captured in traps in various types of ecological habitats (a site covered by grassland vegetation within city limits, an unused agricultural meadow, and a fallow land near a mixed forest). The following rodent species were caught: *Apodemus agrarius, Apodemus flavicollis, Microtus arvalis*, and *Myodes* *glareolus*. Additionally, *Ctenophthalmus agyrtes*, *Ctenophthalmus assimilis*, *Hystrichopsylla talpae*, and *Nosopsyllus fasciatus* flea species were identified. The peak of the flea activity was noted in summer months. *C. agyrtes* was found to be the most abundant flea species in eastern Poland, while the greatest numbers of fleas were collected from the rodent species *A. agrarius*.

## 1. Introduction

Fleas (Siphonaptera) are cosmopolitan, secondarily wingless, hematophagous insects with laterally flattened body symmetry. To date, over 2500 species of fleas representing 238 genera have been described worldwide [1]. They infest warm-blooded vertebrates, mainly mammals and, less frequently, birds [2]. Some flea species have a very wide distribution range (e.g., the human flea *Pulex irritans*, the oriental rat flea *Xenopsylla cheopis*) [3,4], whereas other species are closely associated with specific types of habitats, e.g., forests, mountains, or arid areas (e.g., the sand flea *Tunga penetrans*) [5,6]. Flea adult stages usually colonize burrows and nests or the hair of their hosts [7]. To find a potential host, fleas use chemoreceptors located on the antennae, dorsal shield (pygidium), and legs; these insects are especially sensitive to the smell of lactic acid contained in sweat [8] and are also attracted by the smell of the host’s urine, as well as carbon dioxide exhaled by animals [9]. Fleas sense the body heat of the potential host, but escape from bodies of dead animals [10]. Flea species residing permanently in animal burrows and hideouts exhibit negative phototaxis, whereas positive phototaxis is observed in species present on animal bodies and in open spaces, as it helps them locate a new host [11].

Due to their broad host range and the ability to infest different hosts during their lifespan, which is typical for these insects (most species are polyzoic), fleas play an important role as reservoirs of pathogens [12]. Some flea species are vectors of viruses (Myxomatosis virus) [9], bacteria, and rickettsiae, e.g., *Rickettsia typhi*, *Rickettsia felis*, *Bartonella henselae*, *Yersinia pestis*, or *Francisella tularensis* [9,13,14]. The human flea can be an intermediate host for tapeworms *Dipylidium caninum, Hymenolepis nana,* and *Hymenolepis diminuta* [15,16].

Fleabites are manifested by skin lesions, usually red itchy blisters that provoke scratching [17], which may lead to development of secondary bacterial infections that substantially delay the wound healing process [18]. Frequent exposure to fleabites may induce a disease called flea allergy dermatitis, which is frequently diagnosed in dogs and cats. Anemia may develop in extreme cases [19,20].

To date, little research has been conducted in Poland to study the distribution, seasonal activity, and host preferences of fleas. The investigations were mainly carried out in the northern areas of the country, where fleas were collected from rodents trapped in forest areas located along the Baltic coast and on forestless sea dunes [21,22] or from bird nests [23]. The most abundant flea species collected mainly from rodents *Apodemus flavicollis*, *Microtus oeconomus*, and *Myodes glareolus* in this area are *Ctenophthalmus agyrtes*, *Megabothris turbidus*, and *Megabothris walkeri* [21,22]. In the Central Poland Lowlands, which cover the central part of the country, the dominant flea species include *C. agyrtes*, *Ctenophthalmus solutus*, *M. turbidus*, and *Hystrichopsylla orientalis* (most frequently collected from *Apodemus agrarius*, *A. flavicollis*, and *M. oeconomus*) [21,24,25]. A similar species composition of flea fauna is characteristic for the southwestern regions of the country (mainly *C. agyrtes* and *M. turbidus*) [26]. The most common flea species in the mountainous areas in Poland are *C. agyrtes kleinschmidtianus*, *Doratopsylla dasycnema cuspis*, and *Palaeopsylla soricis* collected mainly from *A. flavicollis* and *Sorex araneus* [21]. The few studies conducted thus far in eastern Poland have shown a similar flea species composition and host preferences of these insects to those in the lowland areas of Poland [27]. However, to the best of our knowledge, the available literature provides no data on the seasonal activity of fleas in this area. There are no published results of studies conducted simultaneously in different habitats.

The aim of the present study was to identify the species composition and host preferences of fleas with simultaneous determination of the abundance and rhythms of the seasonal activity of local flea populations present in habitats of eastern Poland (Lublin Province).

## 2. Materials and Methods

### 2.1. Study Area

**Figure 1:**
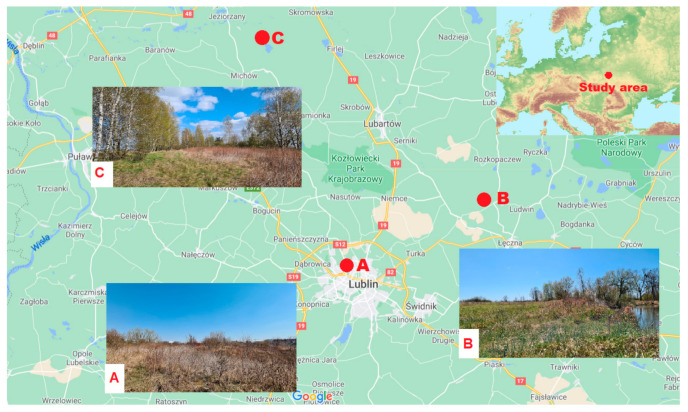
Location of the three study areas A, B and C; (map modified from Google Maps and Wikimedia).

The study was conducted in 3 different ecological habitats in eastern Poland (Lublin Province) in established plots marked with A, B and C (Figure 1).

Plot A (51°16′24″ N; 22°32′04″ E) is located in the immediate vicinity of a residential area within the administrative boundaries of the city of Lublin (approximately 320,000 inhabitants). The area is a recreation site for city residents. It is mostly covered by grassland vegetation with patches of *Solidago virgaurea*, *Tanacetum vulgare*, and *Fragaria vesca*, shrubs *Crataegus oxyacantha* and *Prunus spinosa*, and solitary *Betula* spp. trees.

Plot B (51°21′15″ N; 22°45′39″ E) is part of an unused meadow characterized by progressive ecological succession and located along the Wieprz River, i.e., one of the longest rivers in eastern Poland. The area comprises patches of *Salix* spp. shrubs, and the area surrounding the plot is characterized by an open mosaic-like landscape consisting of sporadically mown meadows and unused crop fields.

Plot C (51°36′41″ N; 22°22′30″ E) is located within a fallow near a mixed forest. It is characterized by clearly progressive ecological succession with predominance of volunteer *Betula* spp. and the presence of solitary *Quercus* spp. and *Pinus sylvestris* trees.

### 2.2. Rodent Trapping Procedure

The number of animals to be captured was based on the 3R principle (refining, reducing, replacing) to limit the number of animals used in the experiment to a minimum ensuring consistent and reliable results.

The rodents were trapped at monthly intervals from May to October 2019. Each time, 40 non-destructive traps (Model S1, R-Max, Mrągowo, Poland) were set up in the plots. The traps were placed at 2-m spacing, thus creating a rectangle with 16 m × 10 m sides and an area of 160 m^2^. Sunflower seeds and carrots were used as bait. Additionally, bearing in mind the possibility of catching small carnivorous mammals, we placed a portion of food of animal origin, i.e., insect larvae (*Zophobas morio*), were placed in each trap. In accordance with the recommendations formulated by Skuratowicz [28], the traps were set up late in the evening and then inspected every 4 h during the day starting from early morning hours. This procedure ensured trapping as many animals as possible, concurrently limiting the number of fleas that had escaped from the host’s body between the subsequent inspections (in fact, this phenomenon cannot be fully eliminated due to the biology of fleas). The rodents were identified to the species level [29]. Specimens of protected species [30] were released into the wild whereas the other animals were killed by dislocation of the cervical vertebrae in accordance with the applicable law (Directive 2010/63/EU of the European Parliament and the Council of 22 September 2010 on the protection of animals used for scientific purposes) [31]. The bodies of the dead animals were disposed of by a specialized company (Bacutil, Zastawie, Poland). 

Due to the seasonality, the field study was conducted from May to October. To prevent hypothermia-induced death of trapped animals, we captured the rodents during the vegetation season on days with a minimum temperature of 10 °C and not in the period with night frosts. Rodent populations have been studied in a similar period of the year in other regions of Poland [21] and in other Central European countries [32]. 

The study was approved by the Local Ethical Committee in Lublin, decision no. 7/28/2018 and the Director of the Regional Directorate for Environmental Protection in Lublin, decision no. WPN.6401.51.2017.MPR.

### 2.3. Identification of Flea Species

Dead animals were placed in plastic sterile 100-cm^3^ containers and kept for maximum 24 h in a portable thermal incubator (BordBar AS 25, Dometic, Stockholm, Sweden) at 5 °C. Next, the ectoparasites were removed from the bodies. For accurate identification and visualization of the details of the morphological structure, we placed the specimens in a 10% KOH solution for 24 h, in accordance with the method proposed by O’Mahony after Sukuratowicz [28]. Next, they were rinsed with distilled water, placed in glacial acetic acid for 15 min, and transferred into xylene for 10 min. Afterwards, the insect bodies were placed in a Petri dish in a drop of 70% ethyl alcohol and the species was identified using a stereoscopic microscope (Stemi 305, Zeiss, Oberkochen, Germany) and an insect identification key compiled by Skuratowicz [28]. After the analyses, the specimens were deposited in the laboratory archives of the Chair and Department of Biology and Parasitology, Medical University of Lublin, Lublin, Poland. 

### 2.4. Analysis of the Quantitative Structure of Flea Populations and Their Host Preferences

The quantitative structure of the fleas was analyzed with the use of the following indices: ecological indices of dominance (*D*%) and index of prevalence (*P*%) [33,34], using the following formulas:

(1) $$D=\;\frac{F_{s}}{F_{t}}\times 100\%$$

where *D*—ecological indices of dominance, $F_{s}$—number of fleas of a given species collected from the rodents, and $F_{t}$—total number of flea species collected from the rodents.

(2) $$\;\;\;\;\;\;\;\;\;\;\;\;\;\;\;P=\;\frac{R_{f}}{R_{t}}\times 100\%$$

where *P*—prevalence, $R_{f}$—number of individual rodent species infested with a particular flea species, and $R_{t}$—number of examined hosts.

The host preferences of the analyzed flea populations were determined on the basis of the indicator proposed by Dudich [35], according to the following formula:

(3) $$I_{p}=\;\frac{N_{f}}{N_{tr}}$$

where $I_{p}$—preference index, $N_{f}$—number of fleas of a given species collected from a given rodent species, and $N_{tr}$—total number of examined rodents. 

### 2.5. Analysis of Weather Conditions

A Data Logger R6030 device (Reed Instruments, Wilmington, NC, USA) for recording of temperature and relative air humidity was placed in each of the plots. The prevailing weather conditions were recorded at 1 h intervals. After completion of the field studies, the data were read and analyzed.

### 2.6. Statistical Analysis

The statistical tests were chosen on the basis of the characteristics of the distribution of the analyzed variables. The relationships between the number of collected females and males of each flea species were tested with the *t*-test for 2 independent means. The seasonal activity of fleas between plots was checked using one-way ANOVA for independent means. The differences in the weather conditions between the analyzed plots were verified with the Kruskal–Wallis test.

In all tests, the value of *p* ≤ 0.05 was considered statistically significant. The statistical analysis was performed using the STATISTICA 10PL package (StatSoft, TIBCO Software Inc., Palo Alto, CA, USA). 

## 3. Results

### 3.1. Occurrence and Seasonal Activity of Fleas

In total, four species of fleas were collected in the study sites. The plots located in the non-urban areas were characterized by greater species richness and abundance of the flea fauna than the plot located in the city (Table 1, Table 2, and Table S1).

*C. agyrtes* was the most numerous flea species. Its representatives were found in each of the plots, and in total 393 fleas were collected from 150 rodents. The ecological index of dominance (*D*%) of this species amounted to 82.91% and was 15.1-fold higher than that in *Hystrichopsylla talpae* and *Nosopsyllus fasciatus* and 13.5-fold higher than in *Ctenophthalmus assimilis*. Moreover, *C. agyrtes* specimens infested 84.0% of all trapped rodents (Table 3). Moreover, 26 specimens of each of the two species *H. talpae* and *N. fasciatus* were collected during the study (the largest number in habitats that had been previously used for agriculture and were then abandoned). *C. assimilis* (in total 29 specimens) was the only species that did not occur in the urban site (plot A) (Table 1, Table 2, and Table S1).

The structure of the local flea populations exhibited a tendency towards a greater number of males than females, but it was not statistically significant in any case (Table 1). 

There were statistically significant differences in the number of fleas collected between the flea populations analyzed (*F* = 17.456, df = 2, *p* < 0.001). Evident rhythms of the seasonal activity of fleas were observed in each plot. The greatest numbers of specimens were collected in June–August, whereas the lowest numbers were found in May (Table 2).

### 3.2. Flea Host Range

**Figure 2:**
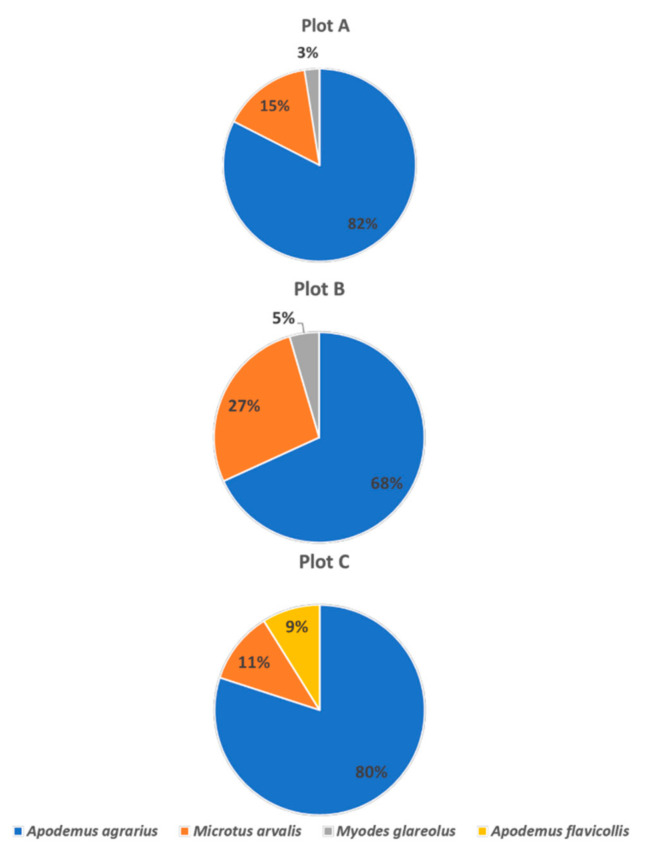
Structure of flea hosts in the study plots.

Flea specimens were most frequently collected from the field mouse *A. agrarius*, which accounted for 68–82% of all captured rodents. The common vole *Microtus arvalis* was another species present in all the habitats. This species represented 11% (fallow near the mixed forest), 15% (urban habitat), and 27% (unused meadow with progressive ecological succession) of the captured animals. Fleas were also collected from the bank voles *M. glareolus* and the yellow-necked mouse *A. flavicollis*; the latter species occurred only in the urban habitat, C (Figure 2, Table 2). 

The highest level of flea infestation was observed in the *M. arvalis* individuals (on average up to 10.0 fleas per animal). The average value of this ratio in the case of the most numerous *A. agrarius* species was 6.2 fleas per animal (Table 2). 

In plot A, the presence of at least one flea species was found in 85.7% of the rodents. This percentage was 97.7% in plot B and 91.0% in plot C. In plot A, higher infestation rates were recorded in *A. agrarius* males than in females. No such a relationship was clearly determined in the other plots due to the high percentage of all infested animals (Supplement Table S1). All the rodent species occurring in the study area should be regarded as the preferred hosts for *C. agyrtes* fleas (Table 4). 

### 3.3. Weather Conditions

Depending on the habitat, the average air temperature measured at the ground level throughout the study ranged from 14.0 °C (plot B) to 17.0 °C (plot A). The mean relative air humidity in this period ranged from 61.1% (plot A) to 77.0% (plot B). 

The analyzed habitats did not differ significantly in terms of the thermal conditions (H = 2.350, *p* = 0.309). However, there was a significant difference in the level of relative air humidity between the habitats (H = 8.995, *p* = 0.011). 

## 4. Discussion

The results of our investigations show that, regardless of the ecological type of the habitat (urban and non-urban habitats), the striped field mouse *A. agrarius* is the most numerous rodent species that can be infested by fleas in the study area (Table 1, Table 2, Table 4, and Table S1). This is associated with the wide ecological tolerance of habitats occupied by the species and the ease of adaptation to changing environmental conditions [36]. 

We observed higher numbers of *A. agrarius* males infested by fleas, in comparison with female individuals. A similar relationship was observed in our previous studies from Lublin [37]. One of the hypotheses elucidating this phenomenon indicates the role of the larger home range of males, which penetrate larger areas; thus, they are exposed to greater risk of attacks by ectoparasites [38]. This may also be associated with the lower immunocompetence in males caused by the immunosuppressive effect of androgens. Furthermore, larger males with higher body weight are a better source of food for parasites and concurrently provide them with a greater variety of niches [39].

In turn, the common vole *M. arvalis* exhibited the highest mean level of infestation per animal (up to 10.0 fleas per individual) (Table 2). The low percentage of this species in the pool of the captured animals (Figure 2), especially in habitats that are preferred by this rodent [40,41], accompanied by the high flea infestation rate may result from the negative impact of the infestation on the animal lifespan [42].

In the study area, we detected the presence of four species of fleas (Table 1). The most abundant species was *C. agyrtes*, which together with *N. fasciatus* is collected most frequently from small mammal species in various parts of Poland [21] and Lublin region [37]. *C. agyrtes* is also one of the most widely distributed flea species in Central Europe [43,44]. This is supported by the lack of clear host specificity [45,46], which was also confirmed by the results of the present study. Specimens of this species were present on 84.0% of all captured rodents and accounted for 82.91% of all collected flea specimens (Table 3 and Table 4). 

The investigated flea populations are characterized by distinct rhythms of seasonal activity. As demonstrated in the most abundant species, i.e., *C. agyrtes*, the peak of flea activity coincided with the peak of the number of rodents (Table 2 and Table S1). This was observed in summer months, when the habitat offers ample food resources for rodents. The results of our study show that enclaves of natural meadow ecosystems with patches of ecological succession provide favorable conditions for sustenance of a stable population of *A. agrarius* and *M. arvalis*. These conditions, together with the presence of rodents, which are a source of food, are also optimal for fleas, which are sensitive to water loss [47]. The low but dense grassland vegetation accumulates water and protects ground surfaces from desiccation, thereby allowing fleas to replenish water. This type of habitat also favors the growth of flea populations, which prefer rodent species that build deep burrows in areas sheltered from sunlight. The insects find both favorable living conditions for adults and an optimal environment for the development of eggs and larvae [48,49,50].

The flea species identified in this study usually infest animals exclusively, except for *N. fasciatus*, which can occasionally attack humans. The epidemiological role of *C. agyrtes*, *C. assimilis*, and *H. talpae* is limited to their involvement as possible vectors of pathogens transmitted between communities of small mammals in the environment and as intermediate hosts for tapeworm larvae [2]. 

## 5. Conclusions

In the area in eastern Poland analyzed in the present study, we found four species of fleas feeding on small rodents: *C. agyrtes*, *C. assimilis*, *H.* *talpae*, and *N. fasciatus. C. agyrtes* was the most abundant flea species identified in the studied area, and the rodents *A. agrarius* were the most preferred hosts of the fleas. The greatest risk of animal infestation by fleas was noted in summer months.

## Figures and Tables

**Table 1:** Occurrence and number of fleas in the experimental plots (F—females, M—males).

Plot	Species	Number of Fleas			Statistics
		F	M	F + M	
A	*C. agyrtes*	48	60	108	*t* = −1.086, *p* = 0.280
	*C. assimilis*	0	0	0	-
	*H. talpae*	4	2	6	*t* = 0.837, *p* = 0.462
	*N. fasciatus*	3	6	9	*t* = −1.044, *p* = 0.299
B	*C. agyrtes*	80	91	171	*t* = −0.767, *p* = 0.444
	*C. assimilis*	9	11	20	*t* = −0.447, *p* = 0.655
	*H. talpae*	5	7	12	*t* = −0.562, *p* = 0.575
	*N. fasciatus*	1	2	3	*t* = −0.581, *p* = 0.562
C	*C. agyrtes*	48	66	114	*t* = −1.740, *p* = 0.846
	*C. assimilis*	6	3	9	*t* = −1.038, *p* = 0.313
	*H. talpae*	3	5	8	*t* = −0.646, *p* = 0.259
	*N. fasciatus*	5	9	14	*t* = −1.104, *p* = 0.272

**Table 2:** Mean level of flea infestation of animals trapped in plots A, B and C (values rounded to 0.1), (N—number of rodents, F—females, M—males).

Plot	Month	Rodent Species	N	Mean Level of Infestation of Rodents (Number of Fleas/Animal)												
				*C. agyrtes*			*C. assimilis*			*H. talpae*			*N. fasciatus*			Total F + M
				F	M	F + M	F	M	F + M	F	M	F + M	F	M	F + M	
A	May	*A. agrarius*	6	0.3	1.3	1.6	-	-	-	0.2	-	0.2	-	-	-	1.8
		*M. arvalis*	1	1.0	-	1.0	-	-	-	-	-	-	-	-	-	1.0
	June	*A. agrarius*	9	1.0	1.2	2.2	-	-	-	0.3	0.1	0.4	0.1	0.1	0.3	2.9
		*M. arvalis*	2	1.5	3.0	4.5	-	-	-	-	-	-	-	-	-	4.5
	July	*A. agrarius*	9	1.5	1.5	3.0	-	-	-	-	-	-	0.1	0.1	0.2	3.2
		*M. arvalis*	1	2.0	2.0	4.0	-	-	-	-	-	-	-	1.0	1.0	5.0
	August	*A. agrarius*	1	2.0	1.0	3.0	-	-	-	-	-	-	-	-	-	3.0
		*M. arvalis*	1	-	3.0	3.0	-	-	-	-	-	-	-	-	-	3.0
		*M. glareolus*	1	2.0	1.0	3.0	-	-	-	-	-	-	-	-	-	3.0
	September	*A. agrarius*	5	1.2	0.3	1.5	-	-	-	-	-	-	-	-	-	1.5
		*M. arvalis*	1	-	-	-	-	-	-	-	-	-	1.0	1.0	2.0	2.0
	October	*A. agrarius*	3	-	1.3	1.3	-	-	-	-	-	-	-	0.3	0.3	1.6
B	May	*A. agrarius*	4	1.7	1.5	-	-	-	-	-	-	-	-	0.2	0.2	1.7
		*M. arvalis*	2	1.5	0.5	2.0	-	-	-	-	-	-	-	-	-	2.0
	June	*A. agrarius*	6	1.2	1.7	2.9	0.2	0.3	0.5	0.2	0.3	0.5	-	-	-	3.9
		*M. arvalis*	4	1.5	-	1.5	0.2	0.5	0.7	0.2	0.2	0.4	-	-	-	2.6
	July	*A. agrarius*	7	1.6	3.3	4.9	0.3	0.4	0.7	0.2	0.2	0.4	-	0.2	0.2	6.2
		*M. arvalis*	2	2.5	3.0	5.2	-	-	-	-	-	-	-	-	-	5.2
		*M. glareolus*	1	2	2	4.0	-	-	-	-	-	-	-	-	-	4.0
	August	*A. agrarius*	8	2.8	3.1	5.9	0.1	0.1	0.2	-	-	-	-	-	-	6.1
		*M. arvalis*	1	4.0	5.0	9.0	-	-	-	1.0	-	1.0	-	-	-	10.0
	September	*A. agrarius*	4	1.8	1.5	3.3	0.2	-	0.2	-	-	-	-	-	-	3.5
		*M. glareolus*	1	2.0	-	2.0	-	-	-	-	-	-	-	-	-	2.0
	October	*A. agrarius*	1	2.0	2.0	4.0	1.0	-	1.0	-	-	-	-	-	-	5.0
		*M. arvalis*	3	1.3	2.0	3.3	-	-	-	-	-	-	-	-	-	3.3
C	May	*A. agrarius*	7	0.6	07	1.1	0.3	0.3	0.6	-	-	-	-	-	-	1.7
	June	*A. agrarius*	1	1.2	1.4	2.6	0.1	0.1	0.2	0.1	0.2	0.3	-	-	-	3.1
		*A. flavicollis*	1	-	-	-	-	-	-	-	-	-	1.0	-	1.0	1.0
	July	*A. agrarius*	7	1.8	2.1	3.9	-	-	-	0.1	0.1	0.2	0.1	0.2	0.3	4.4
		*A. flavicollis*	1	2.0	3.0	5.0	-	-	-	-	-	-	-	-	-	5.0
		*M. arvalis*	1	2.0	-	2.0	-	-	-	-	-	-	-	-	-	2.0
	August	*A. agrarius*	7	1.0	1.8	2.8	-	-	-	-	0.1	0.1	0.1	-	0.1	3.0
		*M. arvalis*	2	1.5	2.0	3.5	-	-	-	-	-	-	-	-	-	3.5
	September	*A. agrarius*	8	1.1	1.2	2.3	0.1	0	0.1	-	-	-	-	0.5	0.5	2.9
		*A. flavicollis*	2	-	1.5	1.5	-	-	-	-	-	-	-	-	-	1.5
	October	*A. agrarius*	6	0.7	1.0	1.7	-	-	-	-	-	-	0.2	0.3	0.5	2.2
		*A. flavicollis*	1	3.0	4.0	7.0	-	-	-	-	-	-	-	-	-	7.0
		*M. arvalis*	2	-	-	-	-	-	-	0.5	0.5	1.0	0.5	0.5	1.0	2.0

**Table 3:** Quantitative structure of fleas collected from small mammals captured in eastern Poland (*D*—ecological indices of dominance, *P*—prevalence).

Flea Species	*D* (%)	*P* (%)
*C. agyrtes*	82.91	84
*C. assimilis*	6.11	12
*H. talpae*	5.49	11
*N. fasciatus*	5.49	15

**Table 4:** Host preferences of the studied flea populations in eastern Poland (<1–low index of host preferences, >1–high index of host preferences). 1–h

Flea Species	Rodent Species			
	*A. agrarius*	*A. flavicolis*	*M. arvalis*	*M. glareolus*
*C. agyrtes*	2.47	3.00	3.25	2.25
*C. assimilis*	0.21	0.20	0.13	-
*H. talpae*	0.17	-	0.25	-
*N. fasciatus*	0.16	0.20	0.25	-

## Supplementary Materials

The following are available online at https://www.mdpi.com/2075-4450/11/12/894/s1, Table S1: Number of collected fleas in particular plots.

## References

[1] Maleki-Ravasan N., Solhjouy-Fard S., Beaucournu J.C., Laudisoit A., Mostafavi E. (2017). The fleas (Siphonaptera) in Iran: Diversity, host range, and medical importance. PLoS Negl. Trop. Dis..

[2] Bitam I., Dittmar K., Parola P., Whiting M.F., Raoult D. (2010). Fleas and flea-borne diseases. Int. J. Infect. Dis..

[3] Aiguo Z. (2003). The characteristics of geographic distribution of *Xenopsylla Cheopis* and the current of epidemic situation of the plague. Chin. J. Pest. Cont..

[4] Zurita A., Callejón R., García-sánchez Á.M., Urdapilleta M., Lareschi M., Cutillas C. (2019). Origin, evolution, phylogeny and taxonomy of *Pulex irritans*. Med. Vet. Entomol..

[5] Linardi P.M., Calheiros C.M.L., Campelo-Junior E.B., Duarte E.M., Heukelbach J., Feldmeier H. (2010). Occurrence of the off-host life stages of *Tunga penetrans* (Siphonaptera) in various environments in Brazil. Ann. Trop. Med. Parasitol..

[6] Deka M.A. (2020). Mapping the geographic distribution of tungiasis in Sub-Saharan Africa. Trop. Med. Infect. Dis..

[7] Urdapilleta M., Linardi P.M., Lareschi M. (2019). Fleas associated with sigmodontine rodents and marsupials from the Paranaense Forest in Northeastern Argentina. Acta Trop..

[8] Slifer E.H. (1980). Chemoreceptors and Other Sense Organs on the Antennal Flagellum of the flea, *Ctenocephalides canis* (Siphonaptera: Pulicidae). Ann. Entomol. Soc. Am..

[9] Durden L.A., Hinkle N.C., Mullen G.R., Durden L.A. (2019). Fleas (Siphonaptera). Medical and Veterinary Entomology.

[10] Fielden L., Krasnov B., Khokhlova I., Arakelyan M. (2004). Respiratory gas exchange in the desert flea *Xenopsylla ramesis* (Siphonaptera: Pulicidae): Response to temperature and blood-feeding. Comp. Biochem. Physiol. A Mol. Integr. Physiol..

[11] Humphries D. (1969). Behavioural aspects of the ecology of the sand martin flea *Ceratophyllus styx jordani* Smit (Siphonaptera). Parasitology.

[12] Durden L.A., Wills W., Clark K.L. (1999). The fleas (Siphonaptera) of South Carolina with an assessment of their vectorial importance. J. Vector Ecol.

[13] Krasnov B.R. (2008). Functional and Evolutionary Ecology of Fleas: A Model for Ecological Parasitology.

[14] Sherman D.M. (2007). Tending Animals in the Global Village: A Guide to International Veterinary Medicine.

[15] Ramana K.V., Rao S.D., Rao R., Mohanty S.K., Wilson C.G. (2011). Human dipylidiasis: A case report of *Dipylidium caninum* infection from Karimnagar. Online J. Health Allied Sci..

[16] Kandi V., Koka S.S., Bhoomigari M.R. (2019). Hymenolepiasis in a Pregnant Woman: A Case Report of Hymenolepis nana Infection. Cureus.

[17] Youssefi M.R., Ebrahimpour S., Rezaei M., Ahmadpour E., Rakhshanpour A., Rahimi M.T. (2014). Dermatitis caused by *Ctenocephalides felis* (cat flea) in human. Casp. J. Intern. Med..

[18] Juckett G. (2013). Arthropod bites. Am. Fam. Physician.

[19] Elsheikha H.M. (2012). Flea allergy dermatitis: The continued challenge. Vet. Nurs. J..

[20] Cucchi-Stefanoni K., Juan-Sallés C., Parás A., Garner M.M. (2008). Fatal anemia and dermatitis in captive agoutis (Dasyprocta mexicana) infested with Echidnophaga fleas. Vet. Parasitol..

[21] Kowalski K., Eichert U., Bogdziewicz M., Rychlik L. (2014). Differentiation of flea communities infesting small mammals across selected habitats of the Baltic coast, central lowlands, and southern mountains of Poland. Parasitol. Res..

[22] Haitlinger R. (1972). Drobne ssaki bezleśnych wydm nadmorskich i ich fauna pcheł. Przegląd Zool..

[23] Kaczmarek S., Mohr A. (1995). Pchły z gniazd nurogesia [*Mergus merganser* L.]. Wiadomości. Parazytol..

[24] Karbowiak G., Solarz K., Asman M., Wróblewski Z., Slivinska K., Werszko J. (2013). Phoresy of astigmatic mites on ticks and fleas in Poland. Biol. Lett..

[25] Haitlinger R. (2011). Arthropods (Acari, Anoplura, Siphonaptera) of small mammals from Kujawsko-Pomorskie Province. Zesz. Nauk. Uniwersyeteu Przyr. Wrocławiu.

[26] Haitlinger R. (1989). Arthropod communities occurring on small mammals from non-wooded areas of urban agglomeration of Wroclaw. Acta Parasitol. Pol..

[27] Haitlinger R. (2010). Arthropods (Acari, Anoplura, Siphonaptera) of small mammals of Lubelskie Province. Zeszyty Naukowe Uniwersytetu Przyrodniczego We Wrocławiu—Biologia i Hodowla Zwierząt.

[28] Skuratowicz W. (1967). Pchły-Siphonaptera (Aphaniptera). Klucze do Oznaczania Owadów Polski.

[29] Kowalski K. (1984). Klucz do Oznaczania Ssaków Polski.

[30] https://otop.org.pl/wp-content/uploads/2016/04/Rozporz%C4%85dzenie-ministra-srodowiska-w-sprawie-ochrony-gatunkowej-zwierzat-16_12_2016.pdf.

[31] https://eur-lex.europa.eu/legal-content/EN/TXT/PDF/?uri=CELEX:32010L0063&from=EN.

[32] Baláž I., Zigová M. (2020). Flea Communities on Small Mammals in Lowland Environment. Ekológia (Bratisl.).

[33] Schwerdtfeger F. (1975). Ökologie der Tiere. Band III: Synökologie.

[34] Margolis L., Esch G.W., Holmes J.C., Kuris A.M., Schad G.A. (1982). The use of ecological terms in parasitology (report of an ad hoc committee of the American Society of Parasitologists). J. Parasitol..

[35] Dudich A. (1995). Synecological Study of Small Mammals’ Ectoparasites of Spruce Forests in Stredné Beskydy Mts. (West Carpathians). Ph.D. Thesis.

[36] Stanko M. (2014). Striped Field Mouse (Apodemus Agrarius, Rodentia) in Slovakia.

[37] Zając Z., Kulisz J., Woźniak A. (2019). The striped field mouse (*Apodemus agrarius)* as a host of fleas (Siphonaptera) and tapeworms (Cestoda) in suburban environment of Lublin (eastern Poland). Folia Biol. Oecologica.

[38] Vukićević-Radić O., Matić R., Kataranovski D., Stamenković S. (2006). Spatial organization and home range of *Apodemus flavicollis* and *A. agrarius* on Mt. Avala, Serbia. Acta Zool. Acad. Sci. Hung..

[39] Kowalski K., Bogdziewicz M., Eichert U., Rychlik L. (2015). Sex differences in flea infections among rodent hosts: Is there a male bias?. Parasitol. Res..

[40] Jacob J., Manson P., Barfknecht R., Fredricks T. (2014). Common vole (*Microtus arvalis*) ecology and management: Implications for risk assessment of plant protection products. Pest. Manag. Sci..

[41] Canova L. (1992). Distribution and habitat preference of small mammals in a biotope of the north Italian plain. Ital. J. Zool..

[42] Devevey G., Christe P. (2009). Flea infestation reduces the life span of the common vole. Parasitology.

[43] Heglasová I., Víchová B., Stanko M. (2020). Detection of *Rickettsia* spp. in Fleas Collected from Small Mammals in Slovakia, Central Europe. Vec. Borne Zoonotic Dis..

[44] Krasnov B.R., Stanko M., Miklisova D., Morand S. (2006). Habitat variation in species composition of flea assemblages on small mammals in central Europe. Ecol. Res..

[45] Klimpel S., Förster M., Schmahl G. (2007). Parasites of two abundant sympatric rodent species in relation to host phylogeny and ecology. Parasitol. Res..

[46] Vashchenok V.S. (2006). Species composition, host association and niche differentiation in fleas of small mammals in the Ilmen-Volkhov lowland. Parazitologiia.

[47] Fielden L.J., Krasnov B.R., Still K.M., Khokhlova I.S. (2002). Water balance in two species of desert fleas, *Xenopsylla ramesis* and *X. conformis* (Siphonaptera: Pulicidae). J. Med. Entomol..

[48] Knülle W. (1967). Physiological properties and biological implications of the water vapour sorption mechanism in larvae of the oriental rat flea, *Xenopsylla cheopis* (Roths.). J. Insect Physiol..

[49] Krasnov B.R., Matthee S., Lareschi M., Korallo-Vinarskaya N.P., Vinarski M.V. (2010). Co-occurrence of ectoparasites on rodent hosts: Null model analyses of data from three continents. Oikos.

[50] Krasnov B.R., Shenbrot G.I., Khokhlova I.S., Poulin R. (2006). Is abundance a species attribute? An example with haematophagous ectoparasites. Oecologia.

